# Organ-on-a-chip platforms for drug development, cellular toxicity assessment, and disease modeling

**DOI:** 10.55730/1300-0152.2711

**Published:** 2024-06-25

**Authors:** Muhammad Maaz KHURRAM, Göktürk CİNEL, Özlem YEŞİL ÇELİKTAŞ, Erdal BEDİR

**Affiliations:** 1 Department of Bioengineering, İzmir Institute of Technology, İzmir, Turkiye; 2 Department of Bioengineering, Faculty of Engineering, Ege University, İzmir, Turkiye

**Keywords:** Organ-on-a-chip, microfluidics, biomimetics, drug toxicity screening, drug efficacy screening, disease modeling

## Abstract

Organs-on-chips (OoCs) or microphysiological platforms are biomimetic systems engineered to emulate organ structures on microfluidic devices for biomedical research. These microdevices can mimic biological environments that enable cell–cell interactions on a small scale by mimicking 3D in vivo microenvironments outside the body. Thus far, numerous single and multiple OoCs that mimic organs have been developed, and they have emerged as forerunners for drug efficacy and cytotoxicity testing. This review explores OoC platforms to highlight their versatility in studies of drug safety, efficacy, and toxicity. We also reflect on the potential of OoCs to effectively portray disease models for possible novel therapeutics, which is difficult to achieve with traditional 2D in vitro models, providing an essential basis for biologically relevant research.

## 1. Introduction

The field of microfluidic systems emerged as one of the forerunners of biotechnological and biosensor-based research in the last 30 years. George M. Whitesides, one of the pioneers in the field of microfluidics, defined this field as encompassing science and technology intended to process or manipulate small volumes of fluids in micrometer-level settings where channels with dimensions of tens to hundreds of micrometers are utilized, being referred to as labs-on-chips (Whitesides, 2006). Microfluidic devices consist of small microchannels with high surface area and mass transfer (Bange et al., 2005; Wu et al., 2020). Their microchannels help facilitate the required material flow, sustaining the experimental design at hand, and these can vary based on both the structure and the cargo.

In recent years, microfluidic designs have also gained important roles in the health sciences. One type of microfluidic device platform, known as organ-on-a-chip (OoC) and also referred to as a microphysiological system, is used to continuously culture living cells (Bhatia and Ingber, 2014). OoCs have launched a revolution within the biological and medical sciences, offering extensive advantages in research on drug development (Plesniak et al., 2023), disease modeling (Leung et al., 2022; Loewa et al., 2023; Filiz et al., 2024) and personalized medicine studies (Low et al., 2020).

Key achievements leading to early developments in microfluidics can be dated back as far as 1943, when silicone was manufactured by the Dow-Corning corporation using methods proposed by Frederic Kipping (1863–1949), considered the father of silicon chemistry. Moreover, photolithographic concepts for microfluidic device fabrication (i.e. creating minute patterns on a light-sensitive chemical using the ultraviolet spectrum, integrating silicon as a photomask, and preventing the transfer of photons to determine the pattern on the sensitive chemical) were put forward by Lathrop and Nall (Lathrop and Nall, 1959; Manz et al., 1990). Subsequent work concluded with a patent for a method successfully producing patterns on silicon semiconductors in 1964 (Andrus, 1964). These fundamental advancements led to the birth of microfluidics in the 1990s, which continued to build pace over the last decades. Embarking on microfluidic studies, Manz et al. (1990) successfully conceptualized miniaturized total chemical analysis systems (μTAS). In 1998, Whitesides’ group successfully developed polydimethylsiloxane (PDMS)-based soft lithography techniques (i.e. using polymers with low intermolecular forces and a low Young’s modulus and high failure strain to fabricate functional structures such as stamps, molds, or photomasks), which would become the standard method for microfluidic prototyping (Duffy et al., 1998). Over time, more inexpensive techniques for microfluidic prototyping were explored, and Whitesides’ group introduced paper-based microfluidic methods, gaining momentum for new applications in point-of-care diagnostics in low-resource settings (Martinez et al., 2007). Huh et al. (2010) successfully developed a fundamental OoC system mimicking lung function on a biomimetic device and notably representing the alveolar–capillary interface. With these continual advancements, the field of microfluidics could be integrated into the development of new systems.

Over time, OoC systems have thoroughly evolved into complex platforms capable of capturing key aspects of research ranging from drug discoveries and pathophysiological studies to body-based bioprocesses. These platforms are more advantageous than traditional large-scale systems due to their compact nature, with minute sample and reagent volumes being sufficient for high-resolution and sensitive performances (Didier et al., 2023; Garcia et al., 2023; Wright et al., 2023). They also offer the potential for the manipulation of cellular, biochemical, and biomechanical conditions (Whitesides, 2006; Bottaro and Nastruzzi, 2016; Upaassana et al., 2019).

**Figure 1:**
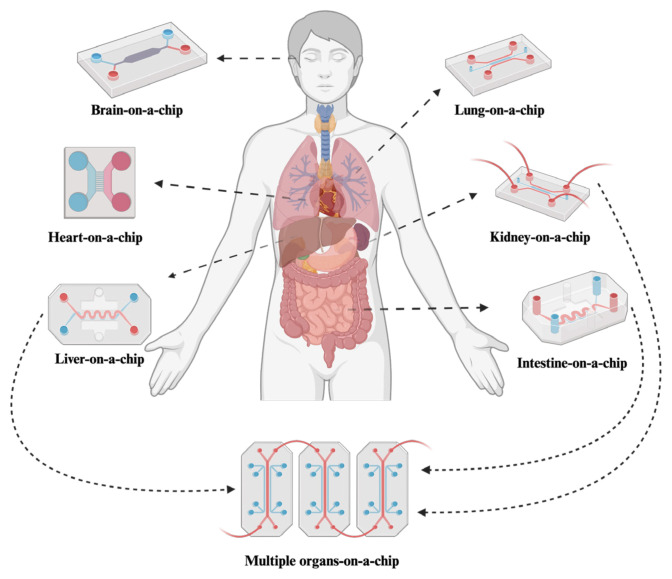
Schematic of microfluidic devices used as OoC platforms showcasing the versatility of microfluidic technology for adapting to diverse organ studies. Created with BioRender.com.

In this review, we present information on microfluidic OoC devices that have evolved as distinct specialized organ chips for drug development, toxicity assessment, and disease modeling, showcasing the versatility of microfluidic devices (Figure 1).

## 2. OoC platforms for the study of drug safety and efficacy

With the onset of new infectious diseases, prevailing deficiencies, hereditary diseases, and increasing drug resistance, it is imperative to pursue new drug discoveries and identify new potential medicines. Circumventing the related obstacles necessitates improving the bioavailability of potential drugs with minimal adverse effects. Traditionally, the determination of a drug’s efficacy and safety profile is a multiphase investigation that requires using groups of animals and, if deemed successful in the first steps, groups of human participants. Undertaking such an exploration is highly risky, and methods for reducing the risks involved must be found to better allocate the limited resources at our disposal. In vivo animal models have been used in drug screening and development studies for years. However, ethical issues concerning animal welfare have led researchers to seek alternate solutions.

Furthermore, in vitro drug testing on human cell lines is considered costly. While providing monolayer cultures, this technique does not replicate the 3D cell structures of in vivo conditions. Differences in the available 2D cell cultures and 3D systems under in vivo conditions create discrepancies in the results of clinical trials. Thus, in vitro drug testing has failed to become a viable alternative to animal use (Ziółkowska et al., 2011). Therefore, a need for novel techniques for drug development and toxicity screenings has emerged.

**Figure 2:**
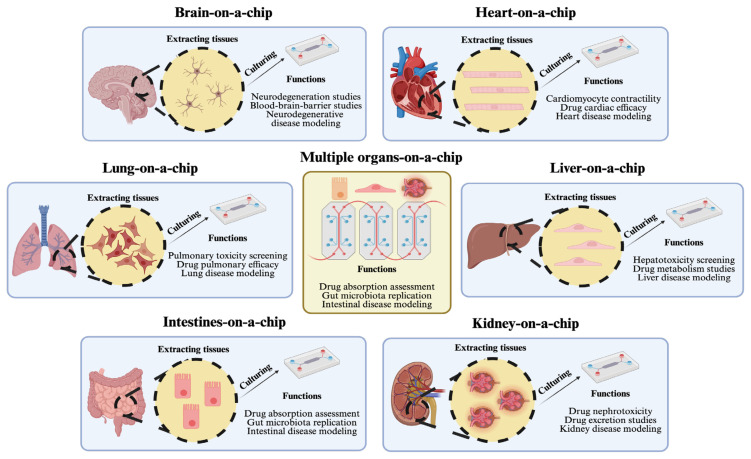
A portrayal of OoC platforms based on their functions and corresponding organs. Created with BioRender.com.

Microfluidic technologies have advanced in the form of platforms offering 3D cell culture mimicking in vivo conditions integrated with microstructures to aid in drug development studies (Cui and Wang, 2019). Many organs can be structured using OoC platforms to study a variety of diseases (Figure 2). The adverse effects of different drug concentrations can also be analyzed. Moreover, low amounts of reagents and samples can be utilized due to the small sizes, minimizing costs and increasing efficiency. Innovations and advantages of microfluidic technologies have also favored the development of OoC platforms for drug discovery and screening studies. OoC models have undoubtedly contributed to a cost-effective in vitro environment (Ma et al., 2021) for pursuing such studies (Table).

Microfluidic systems have proven their worth as promising tools to assess the safety profiles (i.e. assess adverse effects such as cellular toxicity) and efficacy of drugs of interest (Yildiz-Ozturk et al., 2017). Using such systems, researchers can now replicate biological environments and put drug candidates to a preliminary test with levels of physiological relevance that continue to grow. Since an essential percentage of multiphase drug studies tend to fail in any given phase, the initial elimination that might be achieved with microfluidic testing has encouraged researchers worldwide to utilize microfluidic technology before progressing further to clinical studies (McMillan et al., 2016; Syama and Mohanan, 2021; Natu et al., 2023). In the following subsections, we will give examples of how microfluidic methods have helped researchers across the globe gain an understanding of the efficacy and toxicity profiles of drug candidates.

### 2.1. OoC platforms in the study of drug safety

One of the first designs of an OoC model for drug discovery and screening was reported by Grosberg et al. (2011). A heart-on-a-chip model was designed to mimic heart functions using real-time data collection and pharmacological studies with muscular thin film (MTF) technology. Myocytes were organized into anisotropic tissues, rat myocytes were seeded onto fibronectin-bricked walls patterned onto the PDMS surface, and well-established 2D cell culture techniques were implemented. The researchers were able to recreate heart functions with small tissue strip structures. Through the alignment of those small strips, beating was observed under certain conditions with the heart-on-a-chip platform generated with multiple tissues. A fundamental application of this OoC platform entailed conducting pharmacological studies demonstrating the contractile functions of the aforementioned multiple tissues in drug dose–response experiments with epinephrine, a drug that increases cardiac output. As a result of epinephrine treatment, MTF bending was observed due to the spontaneous contractions of cardiomyocytes. A fourfold increase in the strip beating rate was observed as epinephrine concentration increased. This heart-on-a-chip platform recreated heart functions and was used to study appropriate doses for epinephrine treatments (Grosberg et al., 2011).

Another study on myocardium tissue contractility was conducted by the same research group (Agarwal et al., 2013). In that study, the researchers enhanced the MTF technology formerly developed by Grosberg et al. (2011) with entirely autoclavable materials to investigate the effect of isoproterenol, a nonselective beta-adrenergic agonist, on myocardium tissues. A heart-on-a-chip system with MTF was fabricated using laser ablation methodology, coating the cut film with PDMS and seeding muscle cells onto the device. The subsequent recording of myocardium contractility was done by placing the film into a hollow aluminum chamber. The study was designed to assess the contractility of the tissue in a dose-dependent manner, ranging from 1 nM to 100 μM. Control experiment results were obtained from drug-free MTF, while the experimental tests were performed with 10-fold increments of drug concentrations. Myocardium tissue contractions were calculated for four contraction cycles per drug dose. As a result, the researchers captured the twitching in the tissues while using the designed platform and successfully reported the induction effect of isoproterenol on myocardium tissue contraction (Agarwal et al., 2013).

Huh et al. (2010) put forward a lung-on-a-chip microdevice that mimicked lung function in the context of the alveolar–capillary interface. Microchannels in this pioneering OoC platform were fabricated using soft lithography with separation by a flexible PDMS membrane coated with a layer of extracellular matrix (ECM). Human alveolar epithelial cells and pulmonary microvascular endothelial cells were cultured on the opposite sides of that coated membrane. The researchers successfully developed a breathing lung microdevice portraying breathing-type movements using two more lateral microchambers incorporating subatmospheric pressure-driven stretching phenomena. When a vacuum was introduced into these chambers, elastic deformations occurred between the cell-cultured channels and the side chambers. Hence, the PDMS membrane coated with ECM materials started to stretch. When the vacuum was released, the PDMS membrane and cultured cells in the cell culture chamber returned to their original shapes, resulting in breathing-type movements by the lung-on-a-chip microdevice. This pioneering lung-on-a-chip model utilized by Huh et al. (2012) was later upgraded to utilize its potential as a disease model. A human pulmonary edema disease model was established to explore vascular leakage induced by interleukin (IL)-2 in cancer patients and investigate its potential use as a testing platform for new therapeutics against pulmonary edema. Vascular leakage was generated by applying suitable IL-2 doses and subsequently confirmed by phase-contrast microscopy, which showed liquid leakage from the endothelium cell compartment to the air-filled chamber. This upgraded disease model exhibiting vascular leakage was later utilized to identify pharmacological agents preventing IL-2-induced leakage. Angiopoietin-1 (Ang-1) and a transient receptor potential vanilloid 4 (TRPV4) inhibitor (GSK2193874) were identified as potential therapeutics to prevent IL-2-induced vascular leakage. The effects of Ang-1 coadministrated while IL-2 was present in the channel included the inhibition of IL-2-induced vascular leakage, a promising lead for the discovery of therapeutics for human pulmonary edema. This early innovation of a lung-on-a-chip model integrated with a disease model was a key step toward pharmacological modulation for drug discovery and subsequent screenings (Huh et al., 2012).

Another important example of OoCs is gut-on-a-chip platforms. Since the etiological paradigm of systemic diseases started to shift toward a focus on gut axes in the human body, microfluidic studies have tried to mimic the intestinal environment with high accuracy. The journey of creating the most intestine-like environment possible started a decade ago with the establishing of the first gut-on-a-chip to mimic the peristaltic motion of the intestines (Kim et al., 2012). A year after that, the formation of intestinal villi in a microfluidic environment was induced by applying shear stress and cyclic strain on cultured human intestinal cells (Kim and Ingber, 2013). The induced villi formed a barrier that undertook the intestinal function of determining which molecules would be able to pass. This system was reported to mimic intestinal conditions better when induced in the microfluidic environment compared to traditional alternatives. Moreover, in that study, it was shown that *Lactobacillus rhamnosus*, a commensal bacterial species from the human gut microbiota, could be successfully cocultured with CaCo-2 cells, which was a promising step towards creating a fully functional gut-on-a-chip in the future.

From 2013 to 2019, researchers tried to recreate the intestinal environment to carry out reliable experiments that could successfully mimic the real-life intestinal environment. Jalili-Firoozinezhad et al. (2019) created the first gut-on-a-chip platform that held a complex human gut microbiome in an anaerobic microfluidic environment. Their OoC platform encompassed 200 unique operational taxonomic units from 11 different genera with a Firmicutes-to-Bacteroidetes ratio close to the average physiological ratio. Gut-on-a-chip platforms allowed the researchers to induce the growth of intestinal villi that could hold an abundance of different bacteria with a controlled ratio while mimicking the peristaltic motion of the intestines under normal physiological conditions. In subsequent years, gut-on-a-chip platforms were manufactured in tandem with other OoC systems mimicking other organs that work in harmony with the intestines, such as the liver or kidneys. This enabled the researchers to investigate drug metabolism and safety for associated organs via intestinal absorption (Jalili-Firoozinezhad et al., 2019).

One good example of a multi-OoC system is that created by Jie et al. (2017), which was an intestine–liver–glioblastoma biomimetic system applied to analyze the effects of different therapeutics on glioblastoma. The device comprised two layers of PDMS with a hollow fiber in between them in the shape of a sinusoidal curve, which was used to culture CaCo-2 cells. The bottom layer had two chambers to culture HepG2 cells and U251 glioblastoma cells. The chamber used to culture HepG2 cells was just beneath the hollow fiber used to culture CaCo-2 cells, specifically designed to utilize the diffusion of absorbed molecules by the intestinal cells to reach the hepatic cells. The HepG2 chamber was further connected to the upper chamber in the bottom layer where the U251 glioblastoma cells resided. Both the CaCo-2 cell channel and the HepG2 cell channel had inlets and outlets to increase the control of molecular flow in the system. This design enabled the biomimicry of the intestinal drug absorption of irinotecan (CPT-11), temozolomide (TMZ), and cyclophosphamide (CP) via continuous infusion into the intestinal cell channel. After intestinal absorption and liver metabolism, the introduced prodrugs were converted to active forms and conveyed to the glioblastoma cells via the small channels connecting the hepatic and tumor cell channels. The researchers reported that the combination of CPT-11 and TMZ significantly induced the apoptosis of glioblastoma cells compared to single-dose treatments. Moreover, the system’s reusability enabled the comparison of different combinations of drugs within the scope of the experiment. It was concluded that CPT-11 and TMZ were superior to other combinations with a combination index of 0.137 (Jie et al., 2017).

Another example of an OoC model for drug screening came from the work of Jang et al. (2013). They reported a living model of human kidneys produced with a PDMS microfluidic device mimicking kidney proximal tubule function to evaluate drug nephrotoxicity in a microenvironment more similar to in vivo conditions. This model incorporated fluid shear stress, enhancing its physiological relevance. The kidney proximal tubule-on-a-chip consisted of a luminal channel and an interstitial compartment separated by a porous polyester membrane. The membrane was coated with an ECM protein and type IV collagen to simulate in vivo conditions. Human kidney proximal tubule epithelial cells were cultured on the top side of this central porous membrane, involving culture medium flow for 72 h after the initial culture. More importantly, the interstitial compartment was filled with a culture medium to mimic in vivo 3D structures. This proximal tubule-on-a-chip microdevice was able to replicate average cellular height and other important characteristics with shear stress in a flow environment, confirming that the microengineered device mimicked the microenvironments of human kidneys (Jang et al., 2013).

Moreover, in the same study, cellular functions of albumin reabsorption were achieved with the use of flow. The standard functionalities of this engineered microdevice led the researchers to use it as a model for drug development. The impact of cisplatin, a chemotherapy drug causing renal toxicity, was tested on proximal tubule cells using this model in an improved pathophysiological environment. The findings revealed a decrease in LDH enzyme in living cells compared to static conditions. Moreover, under shear-stress conditions, the researchers focused on recreating the activities of Pgp transporters in mediating the effects of drugs provoking multidrug resistance. The flow environments achieved in this tubule-on-a-chip device constituted a breakthrough for representing Pgp efflux activities more effectively. The system facilitated efficient classification between drug candidates as promoters or inhibitors of Pgp transporters in renal tubule cells. Thus, the researchers devised an efficacious tubule-on-a-chip microdevice representing a flow-based system mimicking the normal functions of human kidney tubules for cancer drug screening (Jang et al., 2013).

OoC platforms have also been considered as models for brain-on-a-chip systems to address the limitations posed by other models (Amirifar et al., 2022). One of the earliest brain-on-a-chip models was reported by Park et al. (2014). A microfluidic device was fabricated with 3D neurospheroids mimicking the in vivo brain microenvironment with an interstitial level of flow. This platform enabled the researchers to investigate the effects of flow on neurospheroid size, neural networks, and neural differentiation. It also enabled the testing of amyloid-β, which contributes to Alzheimer disease, and its toxic effects on the developed neurospheroids, resulting in decreased cell viability and destruction of the neural networks. This work linked biomimetic approaches to establish a 3D brain microenvironment to explore the neurotoxic effects of amyloid-β, showing the pathophysiological features of Alzheimer disease (Park et al., 2014).

Recently, a successful multi-OoC system was designed to predict the pharmacodynamic and pharmacokinetic parameters of drugs within interconnected OoC models of the gut, liver, and kidneys (Herland et al., 2020). The connection between these microengineered gut, liver, and kidney devices utilizing vascular endothelium-lined channels reproduced an appropriate environment for evaluation of the first-pass effect. This resulted in the uneven distribution of drug concentrations within the multi-OoC platform, displaying close resemblance to the conditions of living organs. Drugs were introduced to the system via the utilization of an arteriovenous (AV) reservoir used for drug mixing in blood during circulation. The lined vasculature in the separate organ models and the AV reservoir resulted in the controlled, systematic circulation of drug concentrations among the organs while making it possible to monitor their respective concentrations in plasma and blood. Another critical characteristic of this platform was the utilization of a porous membrane between the independent organ models, allowing drug transfer and mimicking drug kinetics from endothelial to parenchymal tissues in an in vivo environment. This first-pass effect multi-OoC model was used to predict the pharmacokinetics and pharmacodynamics of cisplatin administration (Herland et al., 2020).

Moreover, in the same study, nicotine was introduced into the gut chip segment of the multi-OoC system in an orally deliverable formula with a nicotine concentration of 396 ± 16 μM to test drug activities in the gut, liver, and kidney, respectively. Nicotine transfer into the liver chip was observed from the gut chip and from the AV reservoir to the kidney chip. The pharmacokinetics of nicotine in this multi-OoC system were evaluated in terms of nicotine concentrations in the apical and basal channels of the three linked organ chip models. The researchers confirmed nicotine pharmacokinetics to reflect an increasing gradient from the lumen to the designed chips’ basolateral sides. Furthermore, they used the engineered multi-OoC system to study the pharmacokinetics and pharmacodynamics of cisplatin treatment using bone marrow-on-a-chip instead of the gut chip. This set of pharmacodynamics experiments was conducted by administering cisplatin through the AV reservoir to achieve a setup resembling an intravenous injection. The pharmacokinetics of cisplatin were measured in terms of metabolism and clearance by the liver section of the multi-OoC system, which played an important role in measuring the pharmacokinetics. The pharmacodynamics of the administered cisplatin were also evaluated by observing myeloid toxicity in the cells of the bone marrow chip, with a reduction in immune cells showing the in vivo-like side effects of cisplatin treatments. Overall, this multi-OoC model consisting of gut/bone marrow, liver, and kidney OoCs was deemed suitable for evaluating the pharmacokinetics and pharmacodynamics of drug molecules with good physiological relevance (Herland et al., 2020).

These established OoC models for drug development are not limited to studying pharmacokinetics, pharmacodynamics, and adverse effects in the lungs, kidneys, gut, and bone marrow. Other studies have reported drug effects measured with an intestine-on-a-chip system for testing drug adverse effects (Bein et al., 2018). Interconnected multi-OoC models hold value because they can be engineered to study more complicated routes or the effects of drugs in several organs. Therefore, such multi-OoC models can be used to accelerate drug development studies, obtain crucial information on drugs and their potential toxicities in the human body, and translate more reliable data to clinical settings.

### 2.2. OoC platforms in the study of cellular toxicity

Toxic agents leading to organ abnormalities include certain chemicals (Hodgson et al., 2014), environmental factors (Sears and Genuis, 2012), and various drugs (Guengerich, 2011) negatively affecting the cellular machinery. Cellular toxicity is known to be induced with subsequent disastrous effects such as DNA damage via the overproduction of nitric oxide (NO) and reactive oxygen species (ROS) (Hong et al., 2024). Many factors can cause the overproduction of ROS, but mitochondrial dysfunction is one of the most studied and prevalent reasons for it (Vedi and Sabina, 2016). In brief, DNA damage eventually reaches an irreversible point of cellular apoptosis due to mitochondrial dysfunction, subsequently leading to complete organ malfunction. In the last decade, microfluidic-based OoC models have been used to paint a clearer picture of how cellular toxicity affects organ dysfunction.

The alveolar–capillary device developed by Huh et al. (2010) involving a lung-on-a-chip model was also evaluated for its potential in toxicology applications. The research team investigated the adverse effects of silica nanoparticles inducing oxidative stress responses in the alveolar epithelium based on the model’s breathing pattern. The model was proposed as a cost-effective alternative to animal models for toxicology studies (Huh et al., 2010). More recently, Zhang et al. (2018) employed an improved 3D lung-on-a-chip model to evaluate titanium dioxide (TiO_2_) and zinc oxide (ZnO) nanoparticle effects on pulmonary toxicity leading to cellular demise. Using a 3D Matrigel-filled central channel with cultured human pulmonary alveolar epithelial cells and human umbilical endothelial cells in the two flanking channels formed on the PDMS chip, they mimicked alveolar–capillary barrier functions with significant roles in preventing foreign particle-induced toxicity. Interactions between epithelial/endothelial cells and the central ECM modeled in the fabricated microdevice were used to observe changes in structural features with applications of the nanoparticle toxins to better understand acute pulmonary nanoparticle exposure in human lungs. Epithelial cells were exposed to TiO_2_ and ZnO nanoparticles, resulting in an invasion of foreign toxins into the system. Loss in barrier integrity and production of ROS were observed with the addition of the nanoparticle toxins, while 3D confocal reconstruction fluorescence showed decreased barrier integrity upon adding ZnO nanoparticles as a result of diminished junction protein expression with varying nanoparticle concentrations. Production of ROS was also observed with nanoparticle exposure, and increased ROS production was shown in line with varying nanoparticle concentrations. Subsequently, apoptotic cell death, which ROS inevitably cause when they exceed bearable thresholds, was dose-dependent with the incremental addition of ZnO (Zhang et al., 2018). The microengineered 3D lung-on-a-chip device designed in this study was crucial in guiding further investigations examining organ injuries and nanoparticle-based toxicity that can potentially harm organs in ex vivo environments.

Mitochondrial dysfunction is another consequence of oxidative stress that is instrumental in cellular toxicity. Briefly, mitochondrial outer membrane permeabilization releases proteins, including caspases, inducing apoptotic cell death. In the last two decades, microfluidic technologies have been leveraged to develop OoC models to understand the metabolic pathways leading to cell death (Sung and Shuler, 2009; Clevers, 2016; Kim et al., 2019). To study the real-time metabolic processes of the liver, Bavli et al. (2016) reported a liver-on-a-chip model capable of maintaining 3D spheroids of HepG2/C3A cells for 28 days. Crucial parameters within the framework of this liver model included the uptake of oxygen, glucose, and lactate production, which were observed to have a linear relationship with the culture progression of the spheroids over 28 days. Developing such a liver-on-a-chip model allowed the researchers to study the roles of inhibitory molecules that induced mitochondrial dysfunction and stress in the hepatocellular carcinoma cell line. In this regard, rotenone, a broad-spectrum organic compound, was administered to cause mitochondrial damage in the developed platform. Real-time oxygen and glucose uptake measurements were conducted, and a decrease in viable cells was noticed as a decline in glucose uptake levels occurred. This study constituted a significant step forwards toward the real-time monitoring of small molecule-induced toxicity and stress (Bavli et al., 2016).

Another study that investigated mitochondrial dysfunction was conducted by Krenger et al. (2019) using *Caenorhabditis elegans*. They designed a microfluidic device that could harbor a single nematode and used a dye sensitive to oxygen. The fabricated device consisted of 3 layers: the culture chamber layer was designed with dual-cure thiol-ene-epoxy (OSTE+), known to have low gas permeability, so that little to none of the oxygen species could escape the system before the researchers performed measurements. On top of the culture chamber, there were two layers: a PDMS layer to encapsulate the OSTE+ layer and an extra layer of polymethylmethacrylate due to the high gas permeability of PDMS. With this device, the researchers could monitor the growth of a single nematode from the L4 larval stage and study that nematode for six days. The system also had an inlet and outlet, automatically controlled for feeding and waste removal processes. With this device, the nematode was subjected to carbonyl cyanide-4-(trifluoromethoxy)phenylhydrazone (FCCP), a potent mitochondrial decoupling agent that reduces mitochondrial membrane potential, hence rendering oxidative phosphorylation less effective during ATP synthesis. The basal respiratory rate and FCCP-induced respiratory rate were calculated using the microfluidic system, which revealed a difference of a factor of 2 between the two conditions. The nematode was then subjected to sodium azide, which inhibited mitochondrial respiration and yielded poor oxygen levels as real-time readouts using the fabricated device. Overall, this design enabled the cultivation of a single nematode inside a small culture chamber and the study of real-time mitochondrial metabolism (Krenger et al., 2019).

Real-time monitoring is one of the critical aspects that should be provided by OoCs (Cecen et al., 2023). Substantial defects in cellular machinery, such as persistent mitochondrial dysfunction or overexpression of specific cytokines, might indicate a disease state, and real-time monitoring is the best way to understand the underlying disease progression.

## 3. OoC platforms for disease modeling

The modeling of disease states is crucial in portraying progression and potential ways to treat disease onset. Animals or lower organisms are used to simulate pathological aspects of diseases in humans. Key improvements in the field of microfluidics have broadened the horizons of disease modeling with the aid of OoC models to study pathology, disease progression, and novel therapeutics. Such models have been developed from patient-derived sources (Ingber, 2022), stem cells, and the induction of disease-state phenotypes using the genetic tools at our disposal (Benam et al., 2015).

Wang et al. (2014) developed one of the first heart-on-a-chip platforms to recreate the pathophysiology of Barth syndrome (BTHS). BTHS is a rare childhood genetic condition characterized by an enlarged and weakened heart caused by the depletion of mature cardiolipin and accumulation of its immature form, monolysocardiolipin. Moreover, it is known to arise from a mutation in the tafazzin (*TAZ*) gene. The platform modeled the combination of patient-derived and genetically modified induced pluripotent stem cells to show that the disease is linked to a *TAZ* gene mutation. Using mitochondrial function assays, the researchers assessed the metabolic activities of cells originating from patients with BTHS. These groundbreaking initial ideas for heart-on-a-chip development were essential first steps for evaluating disease models and suggesting new therapeutic avenues for rare pediatric disorders (Wang et al., 2014).

Diabetes mellitus is a severe disease affecting how the body utilizes glucose for energy. Diabetic nephropathy (DN) is a severe complication in 30%–40% of people diagnosed with diabetes, causing end-stage kidney disease. DN causes elevations in blood glucose levels and consequential damage in the renal environment. In this regard, Wang et al. (2017) fabricated the first microfluidic model of DN. A glomerulus-on-a-chip system with PDMS recreating a physiologically relevant glomerular microenvironment was established in their study. This glomerular microenvironment was designed in a setting involving a two-layer compartment system with parallel channels. Parallel channels were designed to deliver glomeruli, the 3D ECM, and culture medium. A capillary lumen for the flow of glomeruli and a collection channel connected through Matrigel for the glomerular filtration barrier filtrate were established in this 3D system, mimicking key kidney elements to allow the study of glomerular filtration. The researchers then conducted tests and found a disease model with a high blood glucose level reflecting mechanisms of DN in the microengineered system. After administration of high amounts of glucose, nephrotic cell viability showed pathological changes in the 3D system. In the presence of high blood glucose levels, there was a higher number of nonviable cells, which made the researchers realize that high blood glucose levels could lead to the apoptosis of glomerular cells. Thus, Wang et al. (2017) constructed filtration barriers with high physiological relevance. ROS production in glomerular cells was concluded to be a key reason for the decrease in the quantity of viable cells.

Moreover, the same researchers evaluated the production of ROS in glomerular cells, confirming that high glucose levels induced ROS production that could be lethal for cells. Another relevant observation was the disruption of endothelial cells in the glomeruli due to high amounts of glucose in the disease model. All of these observations led them to successfully manipulate a DN disease model in their promising 3D-engineered system for potential drug testing using this 3D glomerulus-on-a-chip microdevice (Wang et al., 2017).

Another interesting example of the integration of microfluidic technologies in developing OoC disease models came from the work conducted by Hassell et al. (2017). They reported an orthotopic lung cancer model of nonsmall-cell lung cancer (NSCLC) cells used to recreate cancer growth and provide a therapeutic platform for the treatment of NSCLC with an in vitro microengineered device. NSCLC cells were cocultured with primary alveolar or epithelial cells in the lung-on-a-chip model previously engineered by Huh et al. (2010). Physiological breathing motions were mimicked by the lung chip with the application of cyclic suction to the parallel chambers. Therefore, the chip design offered characteristics of a tumor-based lung model. The effects of that breathing on the behavior of tumors were monitored to understand the changes in breathing patterns in the presence of cocultured cancer cells. Tumor cell migration from epithelial cells to endothelial cells was observed over a culture period of 14 days, resulting in a breathing response that could suppress invasiveness by 50%. Moreover, this lung cancer system was utilized to study and analyze drug responses using tyrosine kinase inhibitor drugs, to which patients with NSCLC are known to respond well. Overall, this microfluidic OoC model for NSCLC was suitable for use with the previously built lung model with the addition of NSCLC cells to recreate a disease model and it was found to be convenient for studying drug responses to analyze human resistance to therapeutics (Hassell et al., 2017).

**Figure 3:**
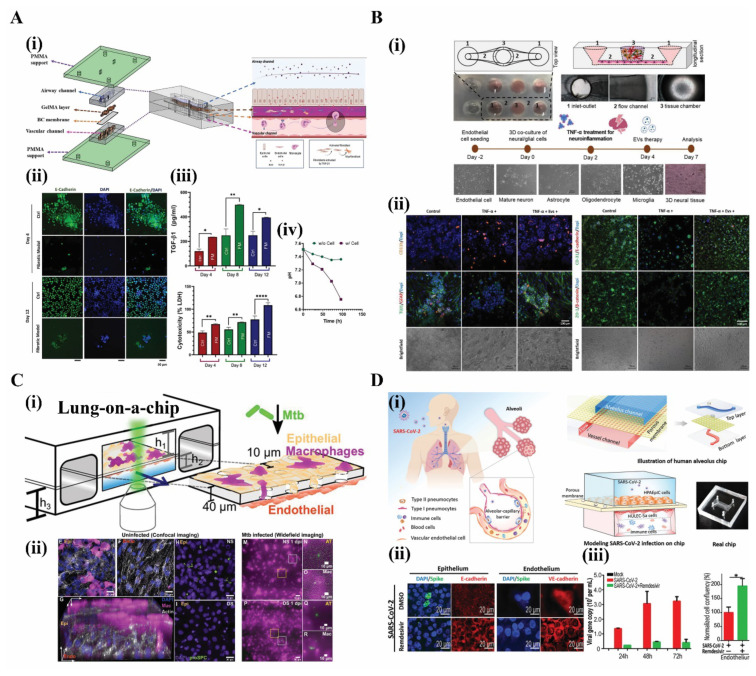
Biomimetic disease model platforms microfabricated as OoCs representing various disease states: **(A)** BLM-induced lung fibrotic model with (i) assembly of a multilayered platform mimicking epithelial injury having a BC membrane and GelMA layer sandwiched between two PDMS layers showing the connection to the interstitial region, (ii) immunofluorescence staining showing the decrease in cell viability in the BLM-induced fibrotic model, (iii) extracellular secretion levels of TGF-β1 and LDH as hallmarks of fibrosis, and (iv) real-time pH measurements with respect to toxicity in the presence of TGF-β1 and LDH (reproduced with permission from Saygili et al., 2023). **(B)** Neural tissue-on-a-chip model portraying TNF-α-induced neuroinflammation with (i) a schematic demonstrating neuroinflammation induced in the neural tissue-on-a-chip model and (ii) bright-field and confocal microscopy images of CD11b and TUJ1/GFAP markers (tissue on-chip construct) and CD-31/E-cadherin and ZO-1/β-catenin markers (blood–brain barrier) after five days of treatment (adapted with permission from Saglam-Metiner et al., 2023). **(C)** Lung-on-a-chip platform to model early tuberculosis (Tb) with (i) a schematic of the early Tb model and (ii) confocal microscopy images of the uninfected lung-on-a-chip model and wide-field microscopy images for the early Tb model (reprinted with permission from Thacker et al., 2020). **(D)** SARS-CoV-2-induced lung model with (i) schematic representation of the human lung-on-a-chip system infected with SARS-CoV-2 with regards to the 3D human in vivo alveolar–capillary barrier, (ii) confocal microscopy images portraying effects of SARS-CoV-2 infection on human epithelium and endothelium, and (iii) efficacy of antiviral drug remdesivir in terms of viral load and epithelial cells treated with or without remdesivir (reprinted with permission from Zhang et al., 2020).

Recently, a lung-on-a-chip model was developed by Saygili et al. (2023) to mimic bleomycin (BLM)-induced pulmonary fibrosis drug-induced lung injuries. The microfluidic platform included a PDMS-based airway and a vascular channel, combined with a bacterial cellulose (BC) layer in between. Human endothelial cells were cultured onto the lower side of the BC membrane, human lung fibroblast cells (CCD-34Lu) cells were cultured in a mixture with a hydrogel-based gelatin-methacrylate (GelMA) layer, and human bronchial epithelium cells (BEAS-2B) were cultured onto the GelMA layer, respectively. This model mimicked the interstitial region of the lungs, displaying the interactions of fibroblasts with the epithelium in the microenvironment (Figure 3A (i)). It also utilized the BC membrane for the first time, as vascular tissue provided a suitable microenvironment for cell interactions and adhesion.

Moreover, this platform integrated a pH sensor to examine pH changes caused by BLM-induced fibrosis. Irreversible epithelial damage was implemented with the addition of 2 μg/mL BLM into the media to recreate pulmonary fibrosis. This was accompanied by a time-dependent decrease in cell growth and a decrease in the levels of epithelial marker E-cadherin in comparison to control groups, showing disturbed epithelial barrier functions and growth patterns according to immunofluorescence staining (Figure 3A (ii)). Fibrosis progression was confirmed by increased levels of TGF-β1 and LDH over a culture period of 12 days in fibrosis models compared to the control experiments (Figure 3A (iii)). BLM-induced fibrosis was also evaluated based on quantitative readouts of pH level changes caused by the pathological and physiological changes occurring within the microfluidic platform. More specifically, pH levels were quantified based on the real-time acidification of media, reflecting profibrotic progression causing LDH-dependent acidification (Figure 3A (iv)). This work paved the way for investigations of FDA-approved antifibrotic drug responsiveness in cases of lung injury while evaluating the ability of the developed platform to model in vitro disease (Saygili et al., 2023).

In another recent report, a neural-tissue-on-chip system was used to evaluate the therapeutic potential of extracellular vesicles (EVs) derived from bone marrow mesenchymal stem cells (BMSCs) to propose treatments for neuroinflammation diseases (Saglam-Metiner et al., 2023). The neural-tissue-on-chip system was established as a blood–brain barrier system supporting 3D neural tissue culture and it consisted of 24 independent culture units with microchannels, divided into two sections by a thin membrane separating the lower culture chamber and open top chamber, respectively. This work addressed the differentiation of mature neurons, astrocytes, microglia, and oligodendrocytes from human induced pluripotent stem cells. Type I collagen was coated onto the surfaces before culturing the human cerebral microvascular endothelial cells (hcMEC/D3) in the microchannels, and mature neurons, microglia, astrocytes, and oligodendrocytes were loaded in the open top chamber in 100 μL of Matrigel matrix. This platform used a 3D neural/glial coculture tissue-on-chip model to mimic the brain tissue and the brain endothelial barrier, respectively (Figure 3B (i)). This tissue-on-chip platform was realized as a neuroinflammation model by supplementing the tissue construct with 50 ng/mL of TNF-α.

Moreover, the effects of EVs derived from BMSCs were highlighted as a treatment option against neuroinflammation. In this regard, BMSC-derived EVs were administered to media at a concentration of 50 μg/250 μL. Reduced cytotoxic effects were observed with decreased TNF-α-mediated LDH activity. The impact of TNF-α effects was also observed on the neural tissue construct, inducing neurotoxic effects after 5 days of TNF-α treatment. Moreover, EV administration enhanced an antiinflammatory response to perpetuate neural tissue construct integrity. EV administration to the inflamed endothelium was also evaluated, and an antiinflammatory response was observed to alleviate neuroinflammation (Figure 3B (ii)) (Saglam-Metiner et al., 2023). This work thus presented a humanized platform for neural-tissue construction that can be applied as a neurodisease model serving as a biomimetic platform for validating novel therapeutics.

Alongside the noninfectious diseases mentioned above, infectious diseases, caused and transmitted by various microbial pathogens such as bacteria, fungi, parasites, and viruses, are another challenge facing the global community. OoC models utilizing state-of-the-art microfluidic technology have provided platforms to model these infectious diseases and perform preclinical drug testing for these infections on engineered devices. A few examples of infectious diseases of great importance around the globe include tuberculosis and the current severe acute respiratory syndrome coronavirus 2 (SARS-CoV-2) virus. With the outbreak of the COVID-19 pandemic, new ideas and research directions were developed regarding disease models for infectious diseases using OoC platforms.

Tuberculosis, one of the most contagious respiratory diseases caused by the bacterium *Mycobacterium tuberculosis*, is a significant public health concern around the globe (Sudre et al., 1992). A murine lung-on-a-chip model of tuberculosis infection was recently developed by Thacker et al. (2020) to recreate the characteristics of the developing infection as a disease model. A PDMS-based lung-on-a-chip system was obtained, having two parallel channels culturing murine epithelial and endothelial cells. Macrophages were also cultured with the epithelial cells, and these two channels were separated by a porous membrane mimicking the alveolar environment (Figure 3C (i)). *Mycobacterium tuberculosis* infection was introduced to the cultured epithelial cells (Figure 3C (ii)). With the successful application of tuberculosis infection by Thacker et al. (2020), this model is crucial for tuberculosis modeling. Other conditions can be incorporated into the system, such as smoking or compromised immune systems, to validate their connections with early and advanced tuberculosis infections without using animals (Thacker et al., 2020).

In another recent report, a novel disease model of SARS-CoV-2 infection was used to evaluate the effects of the virus on lung epithelial and endothelial cells and the possible effects of antiviral drugs on viral load inhibition and the immune response following SARS-CoV-2 infection. The COVID-19 pandemic began in late 2019 when the SARS-CoV-2 virus spread into the human population. Researchers quickly started mimicking SARS-CoV-2 infections with OoC models using refined microfluidic technologies to face this global threat. Zhang et al. (2020) were one of the first research groups to model this infectious disease on such a platform. They designed an alveolus-on-a-chip having two channels separated by a thin, porous membrane coated with a layer of ECM to model the viral infection (Figure 3D (i)). The upper side of the permeable membrane was cultured with human alveolar epithelial type II cells (HPAEpiCs). In contrast, the lower side was cultured with lung microvasculature cells (HULEC-5a), and the vessel channel beneath the membrane was cultured with immune cells to study immune responses after SARS-CoV-2-infection. An ordinary 3D microenvironment of the human alveolus was mimicked adequately using continuously flowing media, fortifying the integrity of the alveolus epithelium–endothelium tissue interface. The integrity of the tissue interface was assessed with E-cadherin staining, leading the researchers to realize that there were adherent junctions identified by E-cadherin staining (Figure 3D (ii)).

Moreover, in the same study, the endothelial cells formed conjunctions revealed by VE-cadherin, confirming an appropriate 3D environment for a human alveolus-on-a-chip model. A disease state model was achieved by infecting epithelial cells with multiplicity of infection of 10. High-resolution transmission electron microscopy and transcriptional analysis revealed that the epithelial cells were more susceptible to the virus than the endothelial cells, and immune responses began in the system 3 days after the infection due to immune cells in the vessel channel. The researchers noted an increased level of inflammatory cytokines, increased recruitment of immune cells, and endothelial cell injury, confirming an immune response to viral infection.

Furthermore, Zhang et al. (2020) used this COVID-19 model to assess antiviral therapeutic drug effects on viral infection. They evaluated the antiviral ability of remdesivir, an antiviral agent used to treat many RNA viruses, against SARS-CoV-2 infection. Remdesivir treatment with an appropriate dose of 10^–6^ M induced suppression of SARS-CoV-2 infection, lessening the alveolar–capillary barrier damage (Figure 3D (iii)). This initial work showed great promise in using a SARS-CoV-2 disease model to implement immune responses. However, using only one type of epithelial cell in a model may not accurately or thoroughly represent the 3D microenvironment of the human alveolus, and only one antiviral therapeutic (i.e. remdesivir) was used. This necessitates the reproduction of the study with various other antiviral agents for a clearer comprehension of the disease pathophysiology (Zhang et al., 2020).

As one of the initial works in the field, the disease model described above showed promising results. It can be improved to develop new antiviral therapeutic agents and variant-specific vaccines with efficacy for novel therapies yet to be discovered in the fight against the COVID-19 pandemic as new virus variants continue to be identified.

## 4. Conclusion

Over the last two decades, OoC models have constituted an emerging field for drug research, cellular toxicity assessment, and disease modeling. OoC platforms have been instrumental, replacing traditional 2D in vitro and in vivo models thanks to their significant advantages. These advantages include utilizing fewer reagents and smaller volumes, requiring less space for bioassays, allowing higher-throughput analyses, and offering increased physiological relevance in modeling 3D biological systems. OoC models have broadened our horizons for conducting high-throughput screening and modeling diseases without ethical concerns. The next step in the field of microfluidics is to develop more precise and accurate systems that can collectively analyze the entire biological machinery of an organism. It is critical to further improve the tools and analytics reviewed in the present study. The field of microphysiological systems can be considered to be in its infancy and requires development in both analytical and engineering fields to reach its full potential in the future.

## Figures and Tables

**Table:** Organ-on-a-chip (OoC) platforms developed to study drug safety and efficacy.

Organ-on-a-chip	Functionality	References
Brain-on-a-chip	Developed a brain-on-a-chip platform with 3D neurospheroids to reveal heightened Alzheimer-related toxicity for improved disease research and drug testing Created 3D brain-like scaffolds for mature human neurons to study Parkinson and Alzheimer diseases, advancing brain-on-a-chip research for disease understanding Created a brain-on-a-chip platform using neural stem cell spheroids to explore how β-amyloid affects neurons and synapses for drug research in the battle against Alzheimer disease Developed a brain-on-a-chip platform that simulates brain networks to study the impact of pridopidine in Huntington disease Generated a 3D neural network using a unique bionanohybrid, neurospheroids, and microelectrode arrays to enable signal transmission and electrophysiological output Crafted a 3D neural tissue-on-chip model to explore therapeutic extracellular vesicles for treating neuroinflammatory disease	Park et al. (2014) Harberts et al. (2020) Liu et al. (2022) Lenoir et al. (2022) Yoon et al. (2022) Saglam-Metiner et al. (2023)
Heart-on-a-chip	Recreated heart functions to investigate the pharmacology of epinephrine and isoproterenol treatments Recreated the physiological and mechanical environment of the heart to assess response to isoprenaline in terms of microtissue contractions Designed heart-on-a-chip model to evaluate cardiac efficacies of verapamil and doxorubicin in decreasing heart rate and beating patterns, respectively Proposed biohybrid hydrogels integrated with microfluidics to recreate a heart-on-a-chip platform for isoproterenol screening Proposed a heart-on-a-chip model mimicking the beating of cardiomyocytes to assess cardiotoxicity and cardioprotective efficacy of clinically approved doxorubicin and cyclophosphamide Proposed a heart-on-a-chip model for long-term dynamic cell culturing to analyze electrically stimulated cardiac tissues in response to verapamil and isoprenaline drug treatments, assessing drug efficacy testing and cardiotoxicity Combined 3D printing with electrospinning technology, fabricating 3D cardiac tissues in a perfusion microfluidic platform to investigate the cardiotoxicity and cardioprotective efficacy of doxorubicin (DOX) and dexrazoxane (DEX).	Grosberg et al. (2011); Agarwal et al. (2013) Marsano et al. (2016) Zhang et al. (2016) Fu et al. (2018) Ren et al. (2020) Liu et al. (2023)
Lung-on-a-chip	Assessed lung breathing functionality to test new therapeutics against pulmonary edema Assessed effects of TiO_2_ and ZnO in pulmonary system toxicity Established a 3D lung-on-a-chip model to assess toxicity in terms of malfunctions of the alveolar–capillary barrier upon PM2.5 exposure leading to pulmonary injury Established a lung-on-a-chip platform to assess the cellular viability of L-cysteine-stabilized ZnO nanoparticles in A549 cells in a perfusion setting Established a lung-on-a-chip model mimicking the microenvironment of lung tissue to study effects of EGFR-targeting drugs gefitinib, afatinib, and osimertinib on tumor cells for drug evaluation and efficacy	Huh et al. (2012) Zhang et al. (2018) Xu et al. (2020) Arathi et al. (2022)
Kidney-on-a-chip	Used kidney proximal tubule functions to study the effects of drug nephrotoxicity Established a multiplexed microfluidic platform to accurately assess kidney toxicity using the *HO-1* gene as a biomarker Established an in vitro microfluidic platform to showcase the effects of cisplatin on cell barrier integrity and transporter function Used a multicompartment kidney-on-a-chip model to replicate drug-induced toxicity on the basolateral membrane, providing high accuracy compared to single-compartment alternatives Examined nephrotoxic molecules (ASO, SPC5001) for 2D kidney cells on a microfluidic platform	Jang et al. (2013) Adler et al. (2016) Vormann et al. (2018) Nieskens et al. (2020) Nieskens et al. (2021)
Liver-on-a-chip	Conducted real-time monitoring of metabolic processes of the liver to investigate small molecule-induced mitochondrial dysfunction and stress Efficiently cultured 1080 cell spheroids, preserving liver function for advanced bioartificial liver development and enhanced drug testing Used microwell liver models combining hepatocyte spheroids and hepatic stellate cells in a chip system to mimic the liver environment, offering improved function and in vitro long-term toxicity testing with acetaminophen and isoniazid Established a liver-on-a-chip platform to enhance liver cell function for drug metabolism studies; statin and its active metabolites were used to validate toxicity in cancer cells Proposed a microfluidic system pairing an albumin sensor with a liver-on-a-chip model that enables continuous monitoring of liver function under drug treatment, offering real-time drug toxicity assessment	Bavli et al. (2016) Ma et al. (2018) Choi et al. (2019) Chen et al. (2021) Asif et al. (2021)
Multiple organs-on-a-chip	Proposed a physiome-on-a-chip system integrating several organ platforms in a single system to enable the clinical translation of preclinical drug discovery and development Connected human bronchial cells and liver spheroids, offering improved assessment of aerosol toxicity of aflatoxin B1 and drug safety for potential lung-targeted medications Assessed ADME properties and cellular toxicity of cisplatin and nicotine administration in a multi-OoC system Established a multi-OoC platform consisting of a liver metabolic model and cancer component to investigate the drug efficacy and inhibitory effects of anticancer prodrug CPT-11, predicting the pharmacokinetics of the drugs via a biomimetic platform Developed a multi-OoC platform to assess the effects of drugs in terms of drug safety and efficacy, evaluating the absorption, distribution, metabolism, and excretion of drugs	Edington et al. (2018) Bovard et al. (2018) Herland et al. (2020) Shinha et al. (2020) Kim et al. (2022)
